# Exogenous Glucose Administration Impairs Glucose Tolerance and Pancreatic Insulin Secretion during Acute Sepsis in Non-Diabetic Mice

**DOI:** 10.1371/journal.pone.0067716

**Published:** 2013-06-24

**Authors:** Yoshio Watanabe, Srikanth Singamsetty, Baobo Zou, Lanping Guo, Darko Stefanovski, Laura C. Alonso, Adolfo Garcia-Ocana, Christopher P. O’Donnell, Bryan J. McVerry

**Affiliations:** 1 Division of Pulmonary, Allergy, and Critical Care Medicine, University of Pittsburgh School of Medicine, Pittsburgh, Pennsylvania, United States of America; 2 Department of Biomedical Sciences, Cedars Sinai Medical Center, University of California Los Angeles, Los Angeles, California, United States of America; 3 Division of Endocrinology and Metabolism University of Pittsburgh School of Medicine, Pittsburgh, Pennsylvania, United States of America; University of Cincinnati, United States of America

## Abstract

**Objectives:**

The development of hyperglycemia and the use of early parenteral feeding are associated with poor outcomes in critically ill patients. We therefore examined the impact of exogenous glucose administration on the integrated metabolic function of endotoxemic mice using our recently developed frequently sampled intravenous glucose tolerance test (FSIVGTT). We next extended our findings using a cecal ligation and puncture (CLP) sepsis model administered early parenteral glucose support.

**Methods:**

Male C57BL/6J mice, 8-12 weeks, were instrumented with chronic indwelling arterial and venous catheters. Endotoxemia was initiated with intra-arterial lipopolysaccharide (LPS; 1 mg/kg) in the presence of saline or glucose infusion (100 µL/hr), and an FSIVGTT was performed after five hours. In a second experiment, catheterized mice underwent CLP and the impact of early parenteral glucose administration on glucose homeostasis and mortality was assessed over 24 hrs.

**Measurements:**

And MAIN RESULTS: Administration of LPS alone did not impair metabolic function, whereas glucose administration alone induced an insulin sensitive state. In contrast, LPS and glucose combined caused marked glucose intolerance and insulin resistance and significantly impaired pancreatic insulin secretion. Similarly, CLP mice receiving parenteral glucose developed fulminant hyperglycemia within 18 hrs (all > 600 mg/dl) associated with increased systemic cytokine release and 40% mortality, whereas CLP alone (85 ± 2 mg/dL) or sham mice receiving parenteral glucose (113 ± 3 mg/dL) all survived and were not hyperglycemic. Despite profound hyperglycemia, plasma insulin in the CLP glucose-infused mice (3.7 ± 1.2 ng/ml) was not higher than sham glucose infused mice (2.1 ± 0.3 ng/ml).

**Conclusions:**

The combination of parenteral glucose support and the systemic inflammatory response in the acute phase of sepsis induces profound insulin resistance and impairs compensatory pancreatic insulin secretion, leading to the development of fulminant hyperglycemia.

## Introduction

Sepsis is a devastating public health problem killing nearly 300,000 people in the U.S. each year [1]. Hyperglycemia frequently complicates this illness imparting a worse prognosis when it develops [2,3]. Optimal regulation of blood glucose in the clinical setting is controversial as several carefully performed large trials have led to mixed conclusions possibly related to differences in patient population, nutritional support, general care protocols, monitoring techniques, and, most importantly, glucose control targets [2,4–8]. What is clear is that hyperglycemia in critical illness portends poor outcome. However, the appropriate level of glucose control remains controversial. Further complicating the issue of glucose homeostasis in critical illness, parenteral nutritional support has been linked to the development of hyperglycemia in critically ill patients [9], and more recently, its early initiation has been associated with increased infection rates, duration of mechanical ventilation, duration of renal replacement therapy, hospital length of stay, and incremental health care costs [8].

Although multiple systems have been implicated in the development of hyperglycemia in critical illness, the basic underlying mechanisms remain poorly understood [10,11]. In addition, the mechanistic relationship between early parenteral nutritional support and adverse outcomes has not been explored. It is generally accepted that hepatic gluconeogenesis is increased and that liver and skeletal muscle become insulin resistant in the septic state [10]. However, our recent data suggest that pancreatic insulin production in response to hyperglycemia may be a key determinant of survival in animal models of sepsis [12]. In contrast to the common clinical observation of hyperglycemia in critically ill adult patients, healthy humans do not become hyperglycemic after exposure to endotoxin, and they demonstrate preserved insulin sensitivity [13–15] suggesting that comorbidities or a combination of insults are required to drive metabolic decompensation in critically ill patients. Similarly, the absence of hyperglycemia is characteristic of multiple animal models of sepsis [12,16–19], and the data presented herein suggest that insulin sensitivity is preserved in endotoxemic mice in the absence of glucose supplementation.

A better understanding of the pathways driving hyperglycemia in the critically ill may allow for the development of novel therapies targeting the mechanisms of metabolic dysfunction rather than the consequences thereof, thus improving outcome while limiting potentially severe adverse effects such as hypoglycemia. We propose that an interaction between circulating glucose and the systemic inflammatory response syndrome propagates hyperglycemia by inducing pancreatic dysfunction in combination with profound insulin resistance in peripheral metabolic tissues.

We recently published the application of the frequently sampled intravenous glucose tolerance test (FSIVGTT) in conscious mice, allowing for the first time an integrated characterization of glucose disposal and concomitant pancreatic function [20]. In addition, we previously reported the observations that hyperglycemia alone is necessary but not sufficient to account for mortality in endotoxemic mice and that profound metabolic dysregulation and death occur when the pancreatic response to hyperglycemia is impaired [12]. We designed the current study to further interrogate glucose and insulin metabolism in endotoxemic mice using the novel FSIVGTT. We examine the hypothesis that circulating glucose and the systemic inflammatory response syndrome combine to drive a cycle of metabolic dysfunction and hyperglycemia in early sepsis, which may contribute to adverse outcome in the setting of early nutritional support. Our results demonstrate that insulin sensitivity is preserved in endotoxemic mice and in mice exposed to exogenous glucose alone, while profound insulin resistance develops in mice exposed to endotoxin in the setting of glucose supplementation. In addition to effects on insulin sensitivity, combined exposure to glucose supplementation and an inflammatory stimulus also impairs pancreatic insulin secretion thereby worsening metabolic dysfunction. Finally, we extend these findings into a clinically relevant sepsis model, cecal ligation and puncture (CLP).

## Methods

### Ethics statement

This study was carried out in strict accordance with the recommendations in the Guide for the Care and Use of Laboratory Animals of the National Institutes of Health. Animal protocols were approved by the Institutional Animal Care and Use Committee at the University of Pittsburgh (Protocol Number: 1106265).

### In vivo endotoxemia

**Figure 1:**
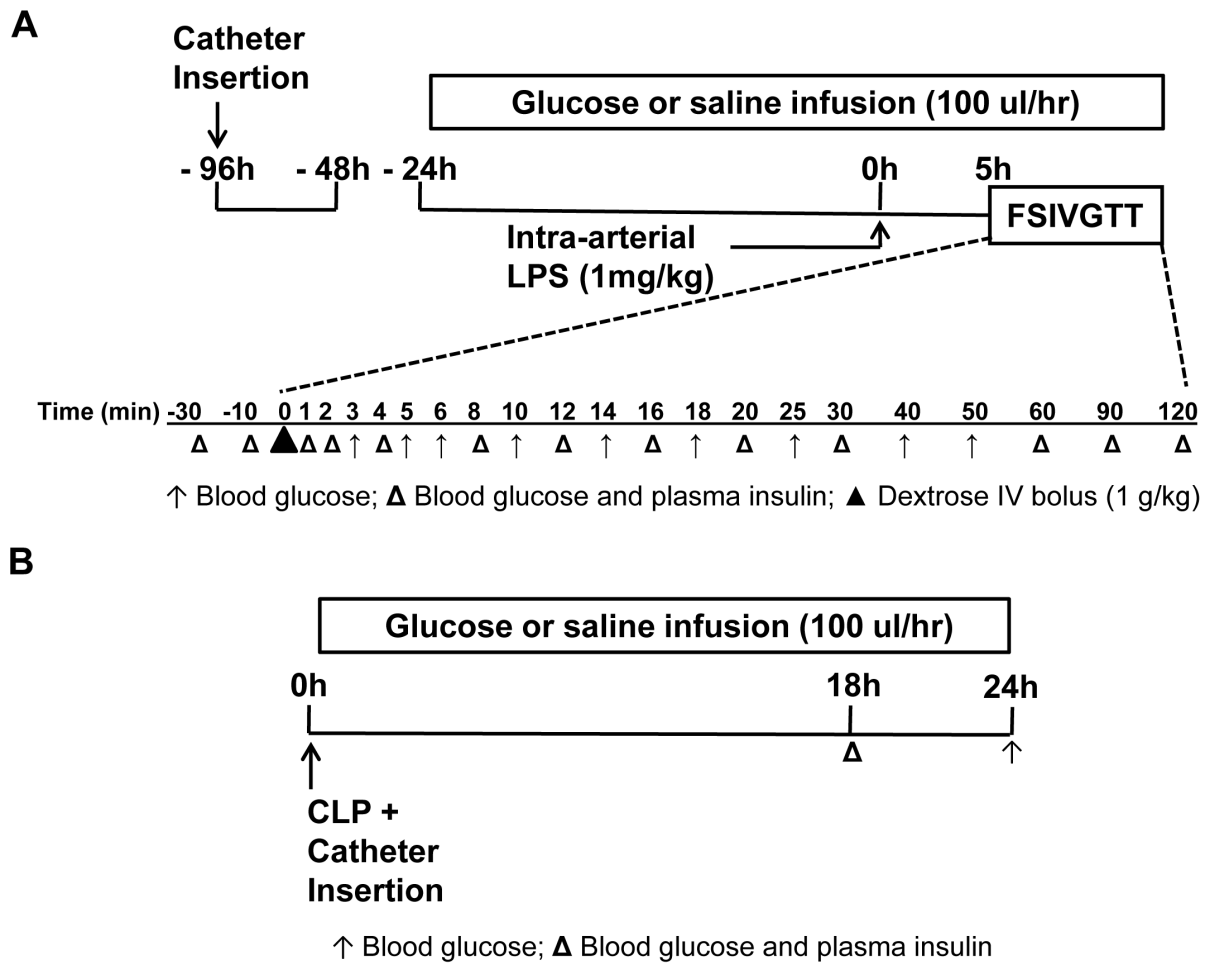
Experimental timelines. (A) Chronically catheterized mice (femoral artery and vein) were given three days to recover from surgery and infused intravenously (iv) with either saline or glucose for 24 hours prior to intra-arterial administration of either lipopolysaccharide (LPS; 1 mg/kg) or vehicle. Five hours after LPS or vehicle administration a frequently sampled intravenous glucose tolerance test (FSIVGTT) was performed over a two hour period. An iv glucose bolus of 1 g/kg D50 was given over approximately 15 sec (▲). Subsequently, multiple samples of either blood glucose and plasma insulin (Δ) or glucose alone (↑) were taken from the arterial catheter at the times identified. Red blood cells were spun down and re-infused into the mouse throughout the protocol to avoid anemia. (B) Mice underwent cecal ligation and puncture (CLP) immediately after chronic catheterization of the femoral artery and vein. Animals were infused intravenously with either saline or glucose upon completion of surgery and followed for 24 hrs. Blood glucose and plasma insulin (Δ) or blood glucose alone (↑) were sampled from the arterial catheter at the times identified.

Male C57BL/6J mice (20–25 g, 8–12 wks old) underwent surgical insertion of indwelling femoral arterial and venous catheters as previously described [12,21]. In brief, mice were anesthetized with inhaled isoflurane (2%) for insertion of micro-renathane catheters (MRE-025; Braintree Scientific, Braintree, MA) into the left femoral artery and vein. Catheters were sutured in place, stabilized with surgical grade tissue glue, tunneled subcutaneously to exit the skin at the upper back, taped to a wire attached to posterior cervical muscles for stiffness (792500; A-M Systems, Sequim, WA), and connected to a 360° dual channel swivel designed for mice (375/D/22QM; Instech, Plymouth Meeting, PA). Catheter patency was maintained by continuous sterile infusion of 7 µL/h saline containing 20 units/mL heparin (APP Pharmaceuticals, Melrose Park, IL) using a syringe pump with multisyringe adaptor (R99-EM; Razel Scientific Instruments, Stamford, CT). Pulsatile blood pressure was monitored continuously, recorded, and stored for off-line analysis, using Windaq Acquisition software (v 2.34, Akron, OH). After three days recovery from surgery, mice began continuous infusions of saline (100 µL/hr) or glucose (100 µL/hr; 50% Dextrose (D50) targeting approximately 40% of daily calorie requirements [21]). Mice had free access to water and chow until five hours prior to the frequently sampled intravenous glucose tolerance test (FSIVGTT). Oral intake was restricted prior to performance of the FSIVGTT as is standard practice prior to detailed metabolic assessment [22]. Endotoxemia was induced 24 hours after infusion initiation by an intra-arterial bolus of bacterial lipopolysaccharide (LPS, 1 mg/kg, *E. Coli* 055:B5 Sigma, Figure 1A) [12]. We chose endotoxemia as a model for sepsis in order to allow for tight control of experimental variables given the complexity of the preparation using chronic double catheterization and the FSIVGTT. We infused glucose for 24 hours prior to inflammatory stimulus in order to maintain consistency with our previously published model [12]. To define the metabolic response of these animals including both peripheral glucose utilization and pancreatic function, FSIVGTT was performed as previously described [20] five hours after LPS infusion (Figure 1A). Briefly, an intravenous (iv) glucose bolus of 1 g/kg D50 was given over approximately 15 sec. Arterial blood was sampled for glucose (~2 µL; glucometer) and insulin (~20 µL; radioimmunoassay) at -30, -10, 0, 1, 2, 4, 8, 12, 16, 20, 30, 6, 90, and 120 min with additional glucose determinations at 3, 5, 6, 10, 14, 18, 25, 40, and 50 min (Figure 1A). Red blood cells were spun down and re-infused into the mouse throughout the protocol to avoid anemia. Heart rate and arterial blood pressure were monitored continuously throughout the FSIVGTT protocol to ensure that the animals were not hypovolemic and maintained stable cardiovascular function. Animals were sacrificed upon termination of the FSIVGTT (t=7hrs) by pentobarbital overdose and exsanguination, according to American Veterinary Medical Association guidelines for euthanasia.

### Mathematical modeling

Insulin and glucose data were entered into a Minimal Model [23] adapted for the faster glucose kinetics seen in conscious mice compared to humans [20]. The Minimal Model provides several key assessments of metabolic function including glucose tolerance (area under the glucose curve, AUC_g_), insulin sensitivity (S_I_), glucose effectiveness (a measure of non-insulin mediated glucose uptake, S_g_), acute insulin response to glucose (an *in vivo* assessment of pancreatic beta cell function, AIR_g_), and the Disposition Index (= S_I_ x AIR_g_), an integrated index of the relationship between insulin sensitivity of the peripheral tissues and compensatory responsiveness of the pancreas (a low disposition index is highly predictive of progression to type 2 diabetes) [23]. Taken together, these parameters provide an integrated *in vivo* assessment of glucose handling and insulin secretion in endotoxemic mice.

### Cecal ligation and puncture

Male C57BL/6J mice (20–25 g, 8–12 wks old) underwent surgical insertion of indwelling femoral arterial and venous catheters as previously described [12]. Immediately following catheter placement, laparotomy was performed and the cecum mobilized. A ligature (3-0 silk) was placed around the proximal 25% of the cecum and tightly secured. A single puncture of the proximal cecum was made with a 16 gauge needle. Fecal matter was expressed by gently squeezing the proximal cecum between the thumb and forefinger, and then abdominal fascia was approximated and the wound closed with surgical grade tissue glue. Control mice underwent sham surgery consisting of laparotomy with manipulation of the cecum and abdominal closure. Immediately post operatively, continuous infusions of saline (100 µL/hr, n=9) or glucose (D50, 100µL/hr, n=10) were initiated (Figure 1B). Oral intake was restricted post-operatively in sham-operated mice in order to control for the absence of oral intake in mice undergoing CLP. Blood pressure and heart rate were monitored continuously, recorded, and stored for off-line analysis using Windaq Acquisition software (v 2.34, Akron, OH). 18 hrs following CLP, blood was sampled from the arterial catheter for insulin, glucose, and cytokine determination as previously described [12]. Surviving animals were sacrificed at 24 hrs. Upon sacrifice, serial dilutions of whole blood were plated, incubated at 37^o^C for 24 hr, and bacterial colonies counted. FSIVGTT was not performed in CLP mice.

### Biochemical Assays

Blood glucose was measured using a Prodigy Autocode glucometer (~1 µL whole blood; Diagnostic Devices, Charlotte, NC). Plasma insulin was measured by radioimmunoassay (5 µL; Linco sensitive rat insulin RIA kit; Millipore, Billerica, MA). Inflammatory cytokines were measured in mouse plasma (5x dilution) using the Bio-Plex Pro mouse cytokine 23-plex assay according to manufacturer instructions (Product # M60-009RDPD, Bio-Rad Laboratories, Hercules, CA).

### Statistics

Data are presented as mean ± standard error of the mean (SEM) unless otherwise indicated. Differences between groups were assessed by one-way or two-way ANOVA where appropriate with post-hoc Bonferroni tests and were considered statistically different for p<0.05. Cytokine data were log transformed for normalization where appropriate prior to statistical analysis. Nonparametric data were analyzed by the Kruskal Wallis test with post-hoc Wilcoxon rank sum tests corrected for multiple comparisons using the Bonferroni method (StataSE v 12.1, StataCorp, College Station, TX). Glucose measurements above the limit of glucometer detection were assigned a value of 600 mg/dL for the purposes of analysis.

## Results

**Figure 2:**
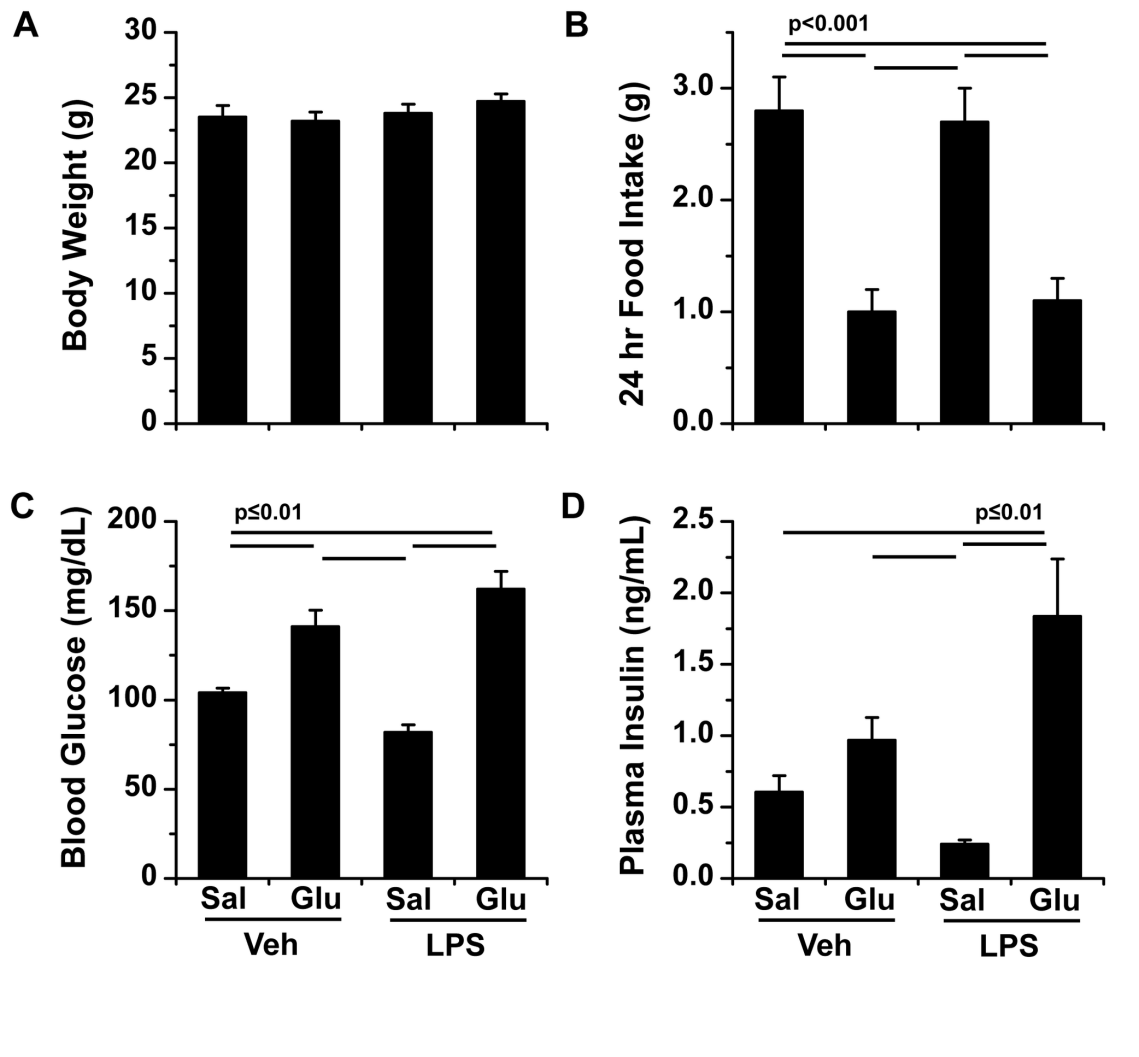
Physiological variables. Mean ± SEM for (A) body weight, (B) food intake over 24 hrs prior to lipopolysaccharide (LPS; 1 mg/kg) administration (note: glucose infused mice consumed fewer calories as the glucose accounted for approximately 40% of their daily calorie intake), (C) pre-FSIVGTT blood glucose, and (D) plasma insulin 5 hrs after either LPS or vehicle (Veh) administration in mice infused with either saline (Sal) or glucose (Glu) at 100 µL/hr for 29 hrs. Statistical differences were determined by two-way ANOVA with differences between individual means determined by post-hoc Bonferroni tests.

A total of 30 mice were catheterized for the endotoxemia study. Experimental groups received saline (Sal) or glucose (Glu) infusion for 24 hrs prior to intra-arterial administration of vehicle or LPS (Veh Sal [n=7], Veh Glu [n=7], LPS Sal [n=8], and LPS Glu [n=8], Figure 1). Five hours after LPS, the FSIVGTT was performed over two hours with saline and glucose infusions maintained throughout (Figure 1). Mice in each of the four experimental groups were of similar weight prior to intervention (Figure 2A). As expected, glucose infused mice consumed fewer calories than their saline infused counterparts (Figure 2B).

Baseline blood glucose and plasma insulin were assessed immediately prior to the FSIVGTT (t=-30 min on Figure 1). Glucose infusion produced a mild hyperglycemia (141 ± 9 mg/dl), whereas LPS administration tended to lower blood glucose during saline infusion (83 ± 5 mg/dl) and raise blood glucose during glucose infusion (169 ± 17 mg/dl) such that baseline blood glucose in the LPS Glu group was double that in the LPS Sal group (Figure 2C 3^rd^ vs. 4^th^ bar, p<0.0001). Baseline plasma insulin followed a similar qualitative pattern as the blood glucose, but insulin levels were 10-fold higher in the LPS Glu group compared to the LPS Sal group (2.30 ± 0.47 vs. 0.22 ± 0.03, Figure 2D, 3^rd^ vs. 4^th^ bar, p<0.0001). These data were consistent with our previously published data [12].

**Figure 3:**
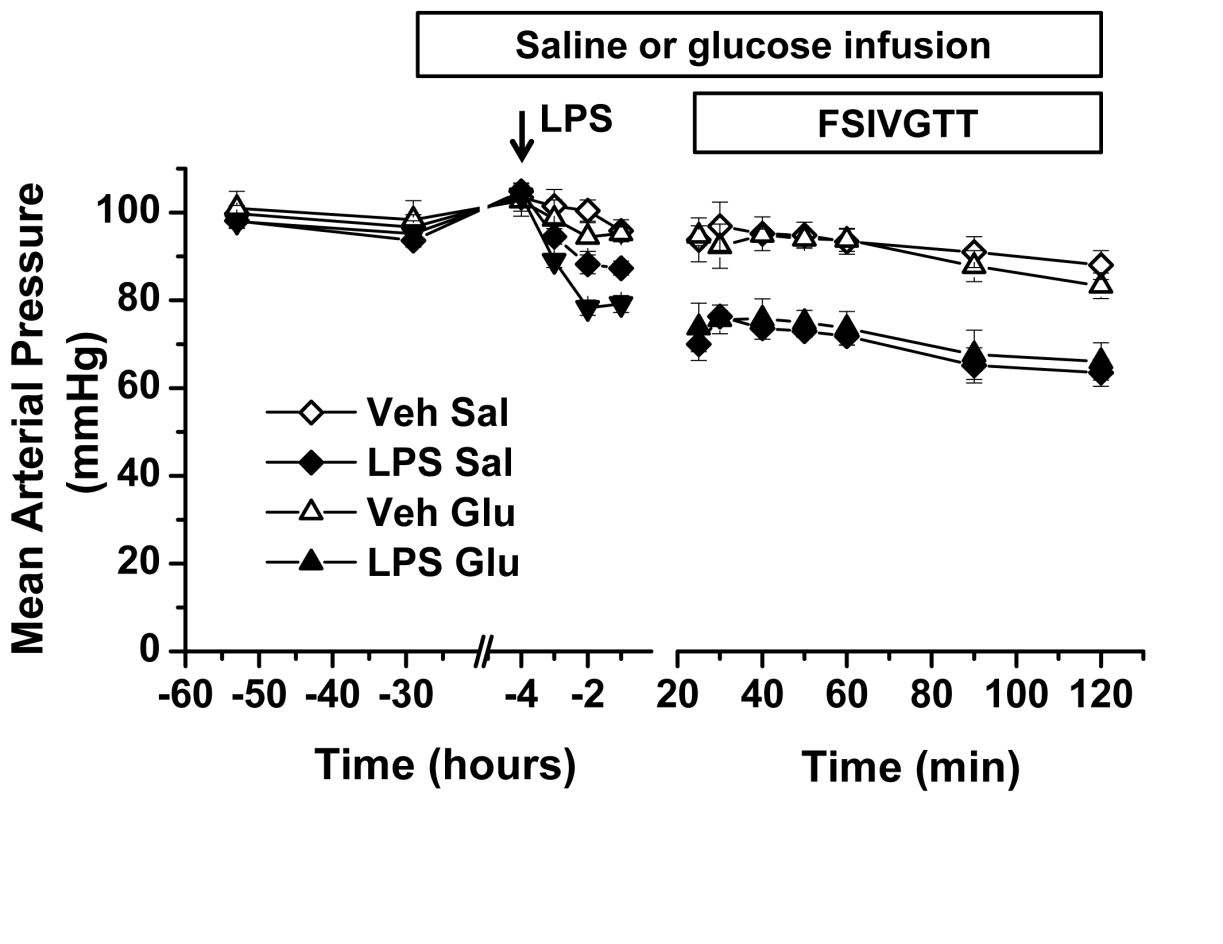
Hemodynamic profiles before and after intra-arterial endotoxin. Change in mean arterial blood pressure over time in four groups of animals receiving either (1) intra-venous saline infusion and vehicle (Veh Sal), (2) intra-venous saline infusion intra-arterial lipopolysaccharide (1 mg/kg, LPS Sal), (3) intra-venous glucose infusion and vehicle (Veh Glu), (4) intra-venous glucose infusion and LPS (1 mg/kg, LPS Glu). Time scale is adjusted to indicate effects of intra-venous saline and glucose infusion, LPS administration, and the frequently sampled intravenous glucose tolerance test (FSIVGTT).

Prior to initiation of and 24 hours into saline or glucose infusion, blood pressure was not different across all study groups (Figure 3). LPS administration induced a drop in blood pressure of approximately 20 mmHg, independent of whether animals were receiving Sal or Glu infusion (Figure 3). Over the two-hour multiple blood sampling period of the FSIVGTT, blood pressure drifted downwards approximately 5-10 mmHg, but did not reach a hypotensive threshold (60 mmHg). The difference in blood pressure between the Veh and LPS groups was maintained through end of the FSIVGTT and remained independent of saline or glucose infusion (Figure 3).

### The combination of endotoxemia and glucose infusion induces profound glucose intolerance

**Figure 4:**
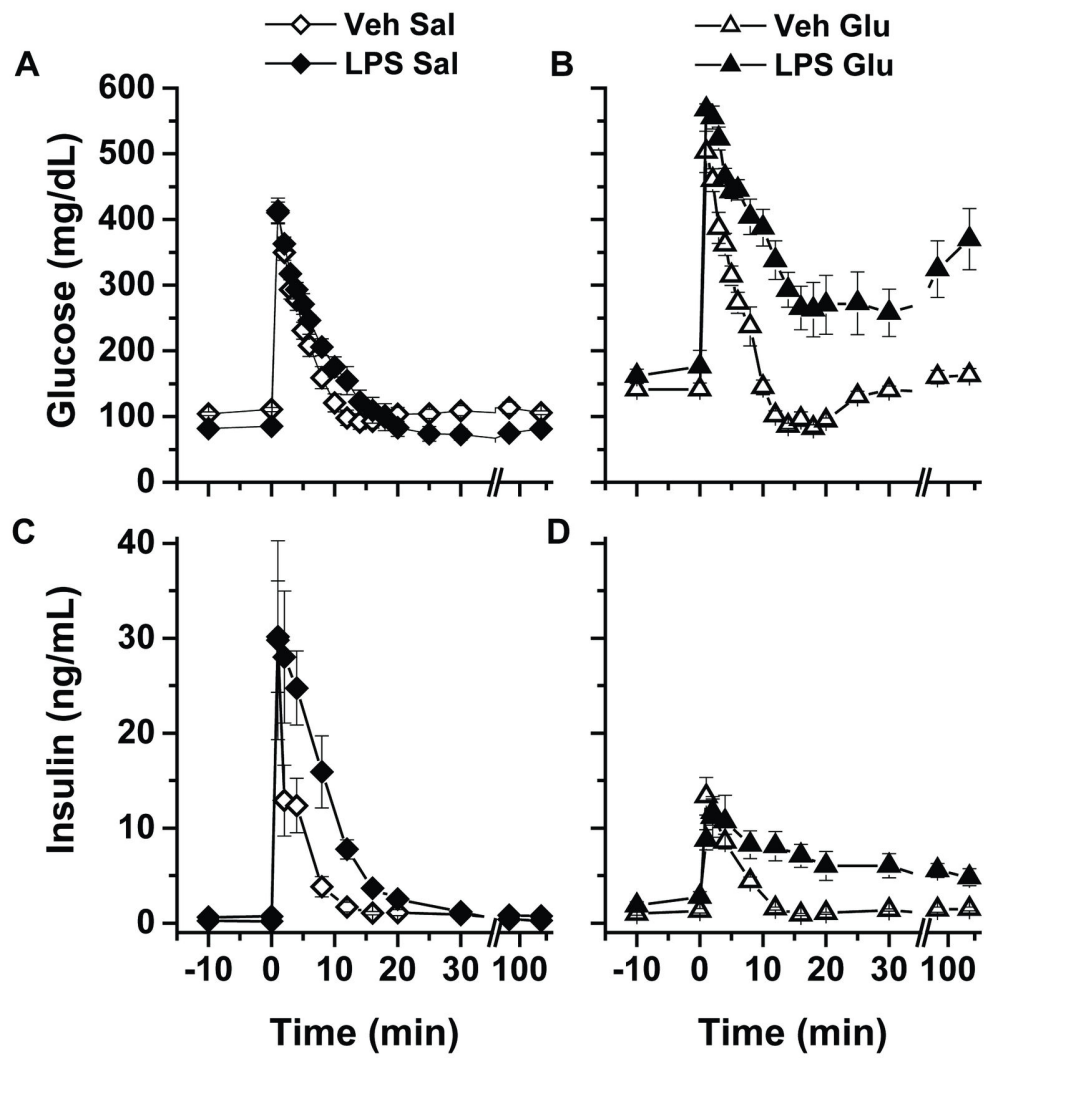
Frequently sampled intravenous glucose tolerance test data. Glucose disposal curves in response to an iv injection of 1 g/kg D50 administered over 15 sec at time t = 0 are depicted for (A) mice receiving iv saline infusion and vehicle (Veh Sal, open diamonds) or iv saline infusion and intra-arterial lipopolysaccharide (1 mg/kg, LPS Sal, closed diamonds) and (B) iv glucose infusion and vehicle (Veh Glu, open triangles) or mice receiving iv glucose infusion and LPS (1 mg/kg, LPS Glu, closed triangles). The corresponding insulin response curves are shown for (C) Veh Sal and LPS Sal and (D) Veh Glu and LPS Glu groups.

**Figure 5:**
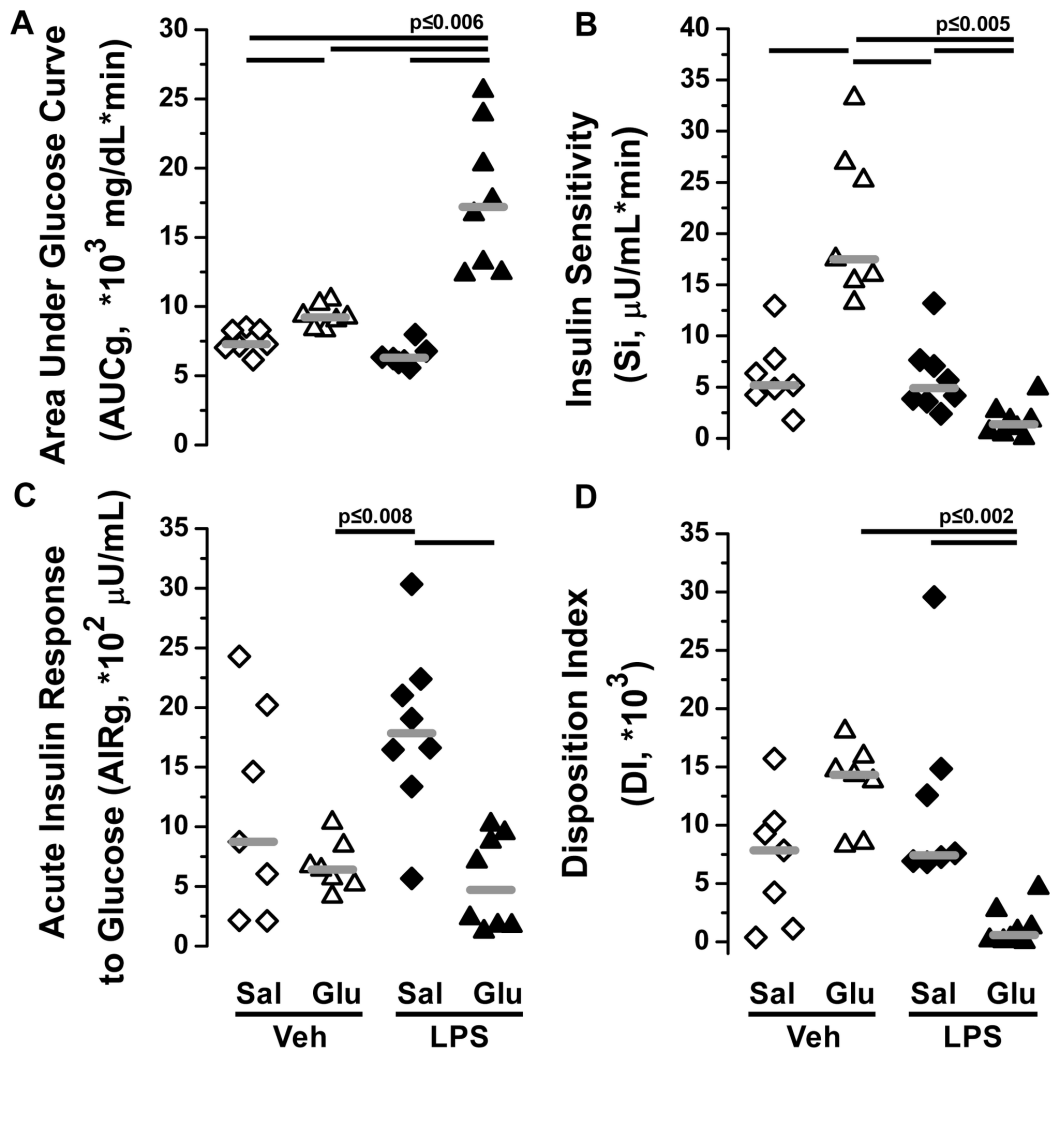
Minimal model data from frequently sampled intravenous glucose tolerance test. Data are shown for (A) glucose tolerance (area under the glucose curve; AUCg), (B) insulin sensitivity (Si), (C) acute insulin response to glucose (AIRg), and (D) Disposition Index (DI = Si *AIRg) for four groups of animals receiving either iv saline infusion and vehicle (Veh Sal, open diamonds), iv saline infusion and intra-arterial lipopolysaccharide (1 mg/kg, LPS Sal, closed diamonds), iv glucose infusion and vehicle (Veh Glu, open triangles), or iv glucose infusion and LPS (1 mg/kg, LPS Glu, closed triangles). Group medians are indicated by gray lines. Statistical differences were determined by Kruskal-Wallis test with differences between individual means determined by post-hoc Wilcoxon rank-sum tests corrected for multiple comparisons using the Bonferroni method.

The glucose disposal and insulin secretion curves for the four experimental groups undergoing FSIVGTT are shown in Figure 4 A–D. Glucose tolerance, defined by area under the glucose disposal curve, was not different between the Veh and LPS groups administered Sal (Figure 4A and Figure 5A, open and solid diamonds). During Glu infusion in the absence of LPS (Figure 4B open triangles), the blood glucose peaked higher than for either of the Sal-infused groups (503±32 [Veh Glu] versus 413±19 [Veh Sal] or 411±16 [LPS Sal] mg/dL, p<0.0001; Figure 4), but due to rapid disposal the glucose tolerance was not different than either of the Sal-infused groups (Figure 5A). Importantly, when LPS was administered in the presence of glucose (LPS Glu), glucose peaked at similar levels to the Veh Glu group (567±9 [LPS Glu] mg/dL), but glucose disposal was markedly impaired (Figure 4B and Figure 5A solid triangles). Thus, whereas we demonstrate a distinct lack of impairment of glucose disposal in response to glucose infusion or endotoxin alone, mice exposed to the combination of LPS and Glu experience profound glucose intolerance.

### The combination of endotoxemia and glucose infusion induces marked insulin resistance and impairs first phase insulin secretion

During saline infusion, the peak insulin secretion was comparable between the Veh Sal (29.8±10.5 ng/ml) and LPS Sal (30.2±5.9 ng/ml) groups (Figure 4C, open and closed diamonds). Although the decay curve for insulin appeared slightly slower for the LPS Sal group than the Veh Sal group, the AIRg were not significantly different (Figure 5C open versus closed diamonds). Similarly, the modeled levels of Si and DI were not different between the saline-infused vehicle and LPS groups (Figure 5B and 5D open and closed diamonds). In both glucose-infused groups, the peak insulin secretion (13.4 ± 2.0 [Veh Glu] and 8.8 ± 1.1 [LPS Glu] ng/mL) was significantly reduced compared to the two saline-infused groups (p = 0.002; Figure 4D). Similarly, the AIRg was significantly less in the glucose-infused compared to saline-infused groups (Figure 5C triangles versus diamonds). The decreased insulin secretion of the Veh Glu group was metabolically appropriate given that a normal DI was maintained by virtue of the development of an insulin sensitive state (Figure 5B and 5D open triangles). However, when LPS was administered in the presence of glucose, the low AIRg was not due to increased sensitivity to insulin; in fact, modeled Si was reduced in the LPS Glu group (Figure 5B closed triangles). The dual metabolic insult of simultaneously reduced Si and AIRg resulted in a DI that was several-fold reduced compared to either the Veh Glu or LPS Sal experimental groups (Figure 5D closed triangles).

There was no evidence that Sg, non-insulin-mediated glucose uptake, was impacted by endotoxemia, glucose administration, or the combination. The Sg in the Veh Sal (0.161 ± 0.037 1/min), LPS Sal (0.093 ± 0.015 1/min), Veh Glu (0.117 ± 0.009 1/min), and LPS Glu (0.140 ± 0.018 1/min) groups were not significantly different from each other. Thus, the changes in glucose disposal that we report are dependent on endogenous insulin secretion and its effect on metabolically active tissue.

In summary, mice exposed to either LPS or glucose infusion alone demonstrate normal glucose tolerance and appropriate pancreatic responsiveness compared to control mice. In contrast, mice exposed to LPS in the setting of glucose supplementation exhibit profound metabolic decompensation characterized by marked glucose intolerance, insulin resistance, and relative pancreatic insufficiency.

### The combination of parenteral glucose with cecal ligation and puncture induces profound hyperglycemia

**Figure 6:**
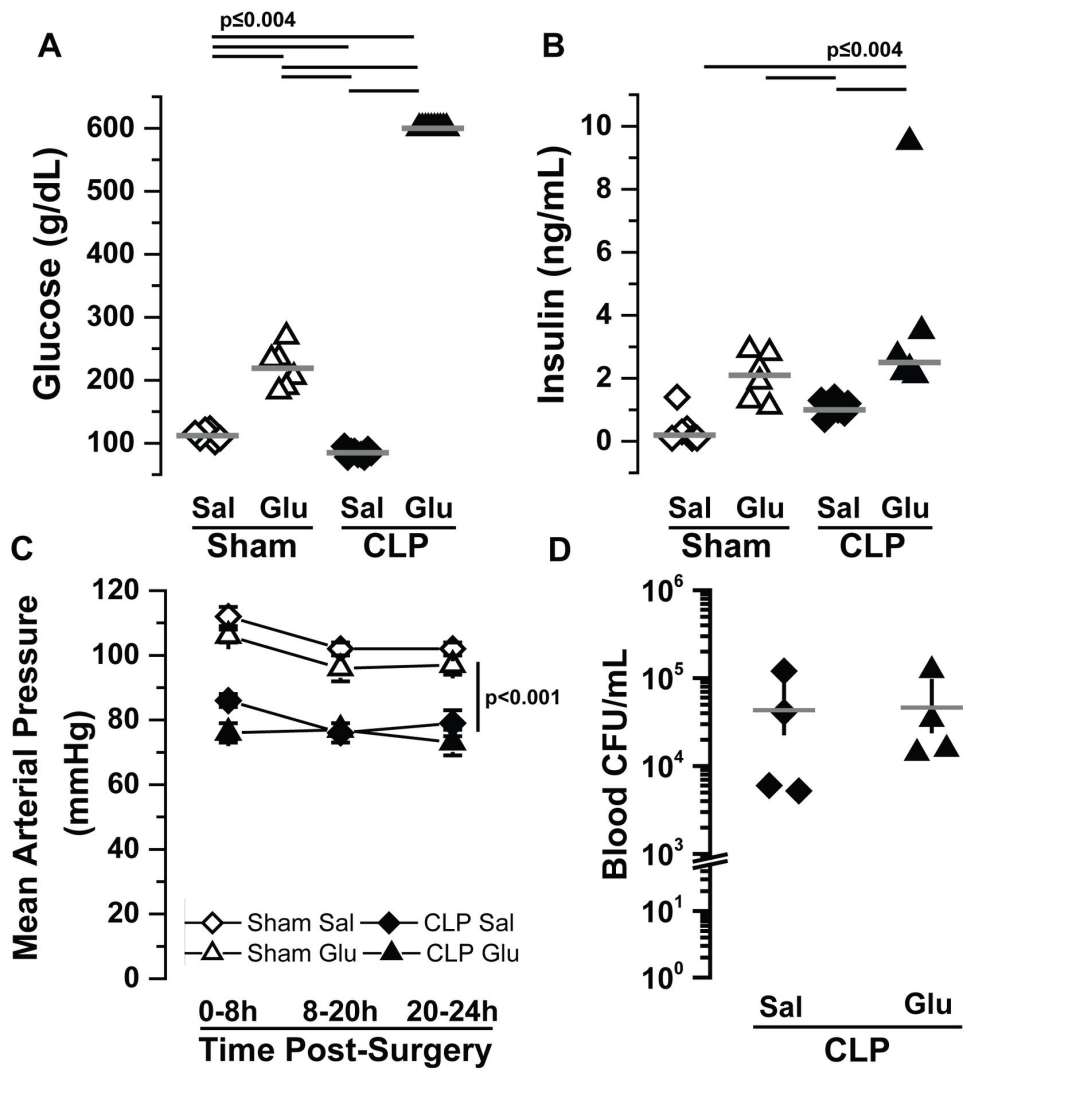
Metabolic and hemodynamic profiles after cecal ligation and puncture. Scatter plot of individual data sets for (A) 18 hr blood glucose, (B) 18 hr plasma insulin and (C) mean arterial blood pressure for mice that have undergone either CLP or sham surgery and infused with iv saline (Sal) or glucose (Glu) at 100 µL/hr for 24 hrs post-operatively (Sham Sal, open diamonds, Sham Glu, open triangles, CLP Sal, closed diamonds, or CLP Glu, closed triangles). Mean arterial blood pressure was averaged over consecutive periods of 8 hrs, 12 hrs, and 4 hrs. Panel D depicts bacterial colony forming units (CFU) in blood extracted from mice 24 hrs after CLP and subject to post-operative saline or glucose infusion. Statistical differences were determined by Kruskal-Wallis test with differences between individual means determined by post-hoc Wilcoxon rank-sum tests corrected for multiple comparisons using the Bonferroni method.

In order to extend the results observed in the endotoxemia model, we employed the cecal ligation and puncture model of sepsis in double catheterized mice. Starting body weights of animals that underwent sham surgery followed immediately with saline were slightly greater than sham animals receiving glucose infusion and those undergoing cecal ligation and puncture (30.5±1.0g [Sham Sal], 26.4±1.2g [Sham Glu], 28.2±1.6g [CLP Sal], 27.6±1.4g [CLP Glu], p<0.0002, n = 6-10 per group). All of the sham animals and CLP Sal animals survived to the completion of the 24 hour study (in our CLP model, mice exhibit 75% mortality at 4 days). In contrast the CLP Glu group exhibited 40% mortality at 24 hours; only data from mice surviving 24 hours were included in the analyses. Similar to the LPS experiments, the presence of sepsis rather than saline or glucose-infusion was the dominant factor impacting blood pressure (Figure 6C). Quantitatively, the approximately 20 mmHg lower blood pressure in the two CLP groups compared to the two sham groups was identical to the blood pressure differences observed between the LPS and Veh groups in the FSIVGTT study (Figures 3 and 6). CLP mice exhibited similar degrees of bacteremia in the presence of either saline or glucose infusion (Figure 6D).

In sham animals administered Glu, both blood glucose and plasma insulin were significantly elevated compared to the Sham Sal group (Figure 6A and 6B open diamonds and open triangles; note that chow intake was restricted in sham mice as CLP mice did not eat post-operatively), replicating the pattern of baseline glucose and insulin we observed in the Veh Sal and Veh Glu groups in the endotoxemia experiments (Figure 2). Again similar to LPS, saline-infused mice undergoing CLP displayed relative hypoglycemia and plasma insulin (Figure 6A and 6B closed diamonds). Strikingly, however, every CLP animal exhibited profound hyperglycemia (> than the 600 mg/dl detection limit of the glucometer) within 18 hours when calories were supplemented with parenteral glucose (note: all the mice from the CLP Glu group not included in the analyses died between 18 and 24 hr after surgery and all exhibited blood glucose > 600 mg/dl at the 18 hr time point). Importantly, the marked hyperglycemia that occurred in the CLP Glu group was associated with plasma insulin levels similar to the Sham Glu group, which exhibited blood glucose levels 2-3 times lower than the CLP Glu group (219±13 mg/dL, p=0.002). Thus, the combination of a systemic inflammatory response syndrome, whether LPS or bacterial in origin, induces marked metabolic dysfunction in the presence of parenteral glucose administration.

### The combination of parenteral glucose with cecal ligation and puncture exacerbates inflammatory cytokine production

**Figure 7:**
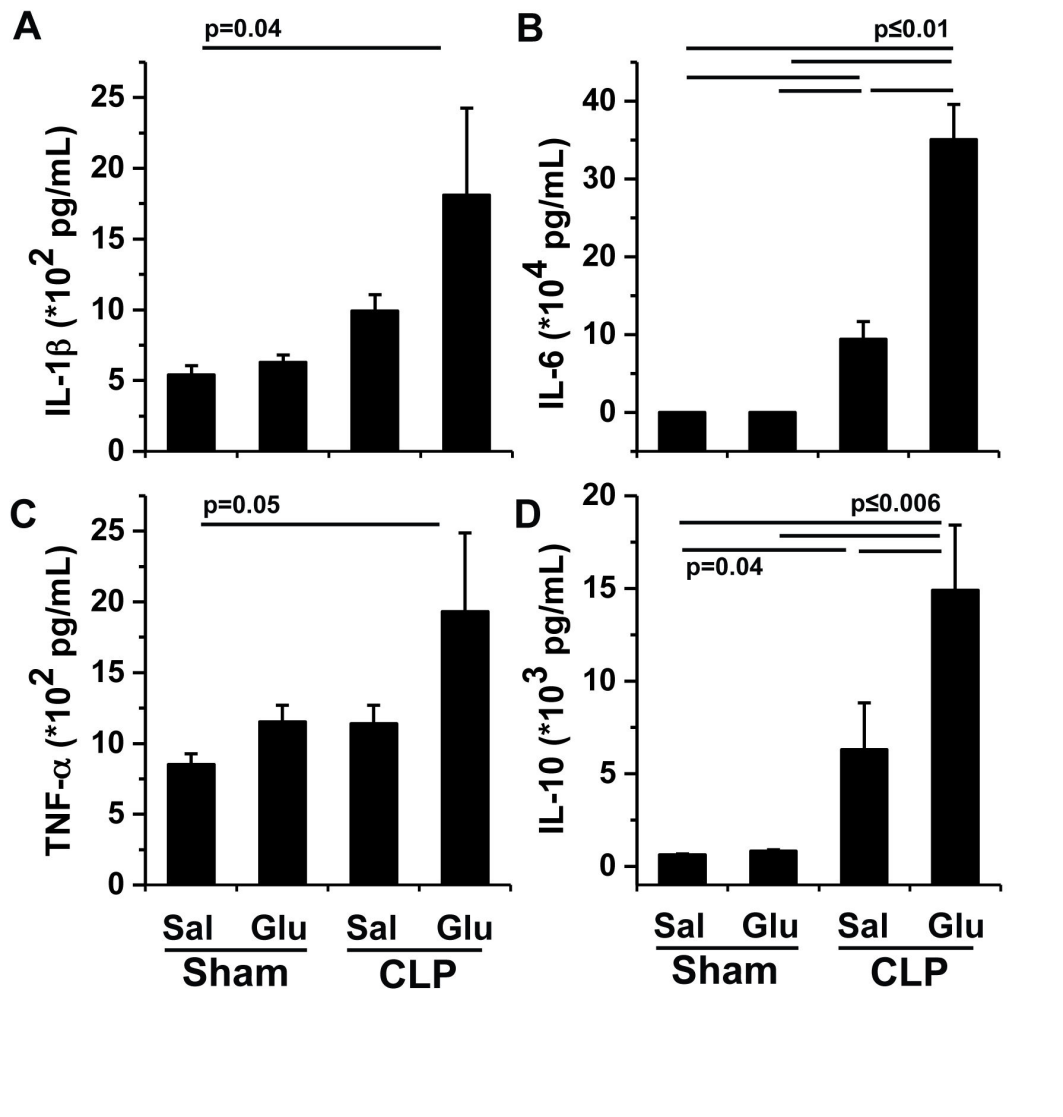
Inflammatory cytokine profiles after cecal ligation and puncture. Mean ± SEM for Interleukin-1β (IL-1β; A), Interleukin-6 (IL-6; B), Tumor necrosis factor-α (TNF-α; C), and Interleukin-10 (IL-10; D) measured in plasma 18 hrs after either CLP or sham surgery and infusion with iv saline (Sal) or glucose (Glu) at 100 µL/hr post-operatively. Statistical differences were determined by two-way ANOVA after log transformation where appropriate with differences between individual means determined by post-hoc Bonferroni tests.

In sham-operated mice, glucose infusion had no effect on circulating Interleukin (IL)-1β, IL-6, tumor necrosis factor (TNF)-α, or IL-10 after 18 hrs (Figure 7 A–D). In CLP mice infused with saline both IL-6 and IL-10 were increased compared to either sham group (Figure 7 B,D). Glucose infusion following CLP (CLP Glu) increased IL-6 and IL-10 compared to saline infusion (CLP Sal, Figure 7 B,D). In addition, CLP Glu mice demonstrated increased IL-1β, IL-6, TNF-α, and IL-10 compared to Sham Sal control mice (Figure 7 A–D). The neutrophil chemo-attractant KC also increased across the four experimental groups in comparable fashion to IL-6 (193±40 pg/mL [Sham Sal]; 189±34 pg/mL [Sham Glu]; 7.7±1.4 x10^4^ pg/mL [CLP Sal]; 34.4±9.9 x10^4^ pg/mL [CLP Glu], p<0.0001).

## Discussion

Previous studies have suggested that nutritional support may be associated with hyperglycemia in the setting of critical illness [9,18,19]. Our study extends these prior observations to demonstrate a novel interaction between endotoxemia and exogenous glucose administration that results in profound insulin resistance and glucose intolerance, and importantly leads to a state of relative pancreatic insufficiency which we have previously shown to be associated with poor outcome in endotoxemic mice [12]. In contrast, neither endotoxemia nor exogenous glucose administration alone negatively impacted metabolic function. The metabolic impairment demonstrated with the combination of endotoxemia and glucose administration occurred with hemodynamics comparable to endotoxemia alone, suggesting that metabolic dysfunction is uncomplicated by circulatory collapse. We extend our observations using an accepted clinically relevant rodent model of sepsis, cecal ligation and puncture, thereby enhancing our understanding of how hyperglycemia develops in critically ill patients. Furthermore, our models demonstrate similar metabolic responses that will allow future exploration of mechanisms underlying the hyperglycemic response and uncovering potential therapeutic targets for correcting dysregulated metabolism in sepsis.

Our prior study focused on early metabolic changes in the evolution of the systemic inflammatory response syndrome using a murine model of endotoxemia. Using this model, we found that suppression of pancreatic insulin secretion negatively impacted glucose regulation and mortality during mild endotoxemia [12]. In order to simultaneously measure insulin sensitivity and insulin secretion in early sepsis we adapted the endotoxin model to include the FSIVGTT. We chose endotoxemia as a model for sepsis in order to allow for tight control of experimental variables given the complexity of the preparation using chronic double catheterization, continuous infusion of substrates, and the FSIVGTT. We extended our endotoxemia results using the cecal ligation and puncture (CLP) model of sepsis, though we were unable to perform the FSIVGTT in these animals as they exhibit profound hyperglycemia in the setting of glucose infusion (beyond the level of detection of our glucometer) precluding the generation of meaningful glucose and insulin disposal curves necessary for modeling of the FSIVGTT. The 24 hr glucose infusion administered in our endotoxemic mice provided a combined enteral and parenteral nutrition model. Our decision to infuse glucose prior to endotoxemia in the FSIVGTT experiments was based on establishing the underlying metabolic abnormalities that occurred in our previous mortality study [12]. Although our model could be interpreted as glucose ‘pre-conditioning,’ our data demonstrate that glucose infused animals in fact display heightened insulin sensitivity and an increased disposition index, indicating these mice were not metabolically compromised. Nevertheless, in the CLP model, infusions were initiated immediately upon completion of the surgery rather than 24 hr in advance as in the endotoxemia study (Figure 1B). This is important in the context of recent human data demonstrating worse outcomes in critically ill patients treated with early intravenous nutrition compared to those in whom nutritional support was delayed and enterally administered [8]. In our CLP model, the mice did not eat and infusion of glucose amounted to approximately 40% of the daily caloric requirement of the mouse, similar to the 20% dextrose infusion provided to the intervention group over the first two days in the clinical study [8]. The glucose dysregulation observed in response to CLP and dextrose administration coupled with our FSIVGTT results provide physiologic insight into the deleterious effect of early intravenous nutrition in critically ill patients whereby glucose infusion in the early stages of critical illness may interact with the ongoing inflammatory response to drive metabolic decompensation and thus poor outcome.

The FSIVGTT has been used to interrogate dynamic insulin secretion and glucose disposal in humans and was first adapted for use in unhandled conscious mice by our group [20]. The FSIVGTT provides an integrated index of metabolism that considers both insulin and non-insulin mediated glucose disposal in the context of insulin secretion from the pancreas, an important consideration that cannot be modeled using other standard methods. Using this novel technique, we identified normal insulin sensitivity in C57BL/6J mice exposed to endotoxin, consistent with data in healthy humans exposed to bacterial endotoxin [14,15]. When combined with glucose infusion, however, endotoxemia dramatically impairs glucose disposal due to marked insulin resistance. The absence of changes in non-insulin mediated glucose uptake between the experimental groups indicates that the impairment in glucose disposal with combined endtoxemia and glucose infusion is specific to either the interaction of insulin with its receptor complex on metabolically active tissue or to one or more components of its well-characterized downstream signal transduction pathway. Using this model, we are unable to determine whether the prolonged elevation in circulating insulin in the endotoxemic mice supplemented with glucose is due to persistent low-level insulin production or delayed clearance. However, our data highlight a striking interaction at the level of the pancreas whereby the insults combine to impede early phase insulin secretion to a level inappropriate for the degree of hyperglycemia, a finding we have previously identified as being critical for survival in endotoxemic mice [12].

A potential limitation of our model is that clinically, hyperglycemia frequently complicates critical illness and our endotoxemic mice without exogenous glucose infusion do not become hyperglycemic, rather they tend to exhibit relative hypoglycemia. This observation is supported both in the animal literature [12,16–18] and in several metabolic studies in endotoxemic humans [13–15]. In order to address this concern and more closely model human sepsis, we adapted the cecal ligation and puncture (CLP) model in our double catheterized mice. Once again, mice became relatively hypoglycemic after septic insult, consistent with our observations in endotoxemia. Notably, mice do not eat after the CLP procedure, while they do take enteral calories after sham surgery, requiring us to restrict access to chow in sham mice to match calorie intake. Importantly, profound hyperglycemia ensued following CLP only in the presence of glucose supplementation, again suggesting a strong relationship between circulating glucose and the development of further metabolic decompensation in sepsis.

While our study does not address mechanism specifically, the notion that glucose supplementation propagates metabolic dysfunction in animals experiencing the systemic inflammatory response syndrome is potentially important as it relates to the care of critically ill patients. Furthermore, the identification of pancreatic dysfunction in these animals may have important translational implications. One attractive potential mechanism driving this pathophysiologic response relates to inflammatory cytokine production. For example, IL-1β has been demonstrated *in vitro* to suppress pancreatic β-cell function and to impair insulin signaling in peripheral tissues [24–26]. Similarly, IL-6 and TNF-α have been shown to suppress insulin signaling and GLUT-4 receptor expression in skeletal muscle, both of which result in insulin resistance [10,11,27]. Our data suggest that the administration of glucose early in the evolution of sepsis can exacerbate the inflammatory cytokine response. The lack of statistical difference between CLP Glu and CLP Sal for IL-1β and TNF-α could be due to (1) the time course of the study as these cytokines are typically produced early following inflammatory insult, or (2) statistical power as our study was designed to show metabolic differences between groups rather than differential cytokine repsonses. Additional mechanisms contributing to hyperglycemia including alterations in glucose delivery due to tissue perfusion, increases in counter-regulatory hormones, changes in lipid profiles, and impaired insulin signaling remain to be explored in CLP mice receiving glucose support.

Glucose metabolism may change with time as sepsis evolves. Our data are limited to the early phase of inflammation as sepsis develops and cannot be extrapolated to later phases of the syndrome, which may display very different metabolic characteristics. Our data are instructive, however, in that they demonstrate that even low level glucose supplementation early in the inflammatory response, such as that provided by the provision of early parenteral nutrition [8] or by maintenance 5% dextrose infusion in a 70 kg human, may be detrimental, triggering a cascade of metabolic derangement that may negatively impact outcomes.

## Conclusions

Glucose control and nutritional support in critically ill patients have been the subject of debate and controversy for the past decade. While the degree of glucose control and optimal caloric supplementation remain unclear, it is generally accepted that hyperglycemia foreshadows poor outcome in critical illness, and recent evidence suggests that early caloric supplementation may worsen outcome [8]. The mechanisms underlying the development of hyperglycemia in sepsis remain poorly defined, and the protective role of endogenous insulin production versus the detrimental role of glucose toxicity continues to be controversial. Our data suggest that a mechanism exists whereby glucose supplementation early in the evolution of the systemic inflammatory response syndrome combines to drive the development of peripheral insulin resistance and suppresses pancreatic insulin production thereby amplifying metabolic decompensation and worsening the degree of hyperglycemia and potentially outcome. Additional investigation is required to explore the mechanism by which circulating glucose and systemic inflammation interact to alter glucose disposal and the pancreatic beta cell response to hyperglycemia in the setting of sepsis.

## References

[1] Angus DC, Linde-Zwirble WT, Lidicker J, Clermont G, Carcillo J et al. (2001) Epidemiology of severe sepsis in the United States: analysis of incidence, outcome, and associated costs of care. Crit Care Med 29: 1303-1310. doi:10.1097/00003246-200107000-00002. PubMed: 11445675. 11445675 10.1097/00003246-200107000-00002

[2] Van den Berghe G, Wouters P, Weekers F, Verwaest C, Bruyninckx F et al. (2001) Intensive insulin therapy in critically ill patients. N Engl J Med 345: 1359-1367. doi:10.1056/NEJMoa011300. PubMed: 11794168. 11794168 10.1056/NEJMoa011300

[3] Frankenfield DC, Omert LA, Badellino MM, Wiles CE 3rd, Bagley SM et al. (1994) Correlation between measured energy expenditure and clinically obtained variables in trauma and sepsis patients. JPEN J Parenter Enteral Nutr 18: 398-403. doi:10.1177/0148607194018005398. PubMed: 7815669. 7815669 10.1177/0148607194018005398

[4] Finfer S, Chittock DR, Su SY, Blair D, Foster D et al. (2009) Intensive versus conventional glucose control in critically ill patients. N Engl J Med 360: 1283-1297. doi:10.1056/NEJMoa0810625. PubMed: 19318384. 19318384 10.1056/NEJMoa0810625

[5] Finney SJ, Zekveld C, Elia A, Evans TW (2003) Glucose control and mortality in critically ill patients. JAMA 290: 2041-2047. doi:10.1001/jama.290.15.2041. PubMed: 14559958. 14559958 10.1001/jama.290.15.2041

[6] Van den Berghe G, Wilmer A, Hermans G, Meersseman W, Wouters PJ et al. (2006) Intensive insulin therapy in the medical ICU. N Engl J Med 354: 449-461. doi:10.1056/NEJMoa052521. PubMed: 16452557. 16452557 10.1056/NEJMoa052521

[7] Brunkhorst FM, Engel C, Bloos F, Meier-Hellmann A, Ragaller M et al. (2008) Intensive insulin therapy and pentastarch resuscitation in severe sepsis. N Engl J Med 358: 125-139. doi:10.1056/NEJMoa070716. PubMed: 18184958. 18184958 10.1056/NEJMoa070716

[8] Casaer MP, Mesotten D, Hermans G, Wouters PJ, Schetz M et al. (2011) Early versus late parenteral nutrition in critically ill adults. N Engl J Med 365: 506-517. doi:10.1056/NEJMoa1102662. PubMed: 21714640. 21714640 10.1056/NEJMoa1102662

[9] Patiño JF, de Pimiento SE, Vergara A, Savino P, Rodríguez M et al. (1999) Hypocaloric support in the critically ill. World J Surg 23: 553-559. doi:10.1007/PL00012346. PubMed: 10227923. 10227923 10.1007/pl00012346

[10] Marik PE, Raghavan M (2004) Stress-hyperglycemia, insulin and immunomodulation in sepsis. Intensive Care Med 30: 748-756. doi:10.1007/s00134-004-2167-y. PubMed: 14991101. 14991101 10.1007/s00134-004-2167-y

[11] Losser MR, Damoisel C, Payen D (2010) Bench-to-bedside review: Glucose and stress conditions in the intensive care unit. Crit Care 14: 231. doi:10.1186/cc9386. PubMed: 20727232. 20727232 10.1186/cc9100 PMC2945096

[12] Woodske ME, Yokoe T, Zou B, Romano LC, Rosa TC et al. (2009) Hyperinsulinemia predicts survival in a hyperglycemic mouse model of critical illness. Crit Care Med 37: 2596-2603. doi:10.1097/CCM.0b013e3181a9338a. PubMed: 19623043. 19623043 10.1097/CCM.0b013e3181a9338a PMC4326234

[13] Agwunobi AO, Reid C, Maycock P, Little RA, Carlson GL (2000) Insulin resistance and substrate utilization in human endotoxemia. J Clin Endocrinol Metab 85: 3770-3778. doi:10.1210/jc.85.10.3770. PubMed: 11061537. 11061537 10.1210/jcem.85.10.6914

[14] Krogh-Madsen R, Møller K, Dela F, Kronborg G, Jauffred S et al. (2004) Effect of hyperglycemia and hyperinsulinemia on the response of IL-6, TNF-alpha, and FFAs to low-dose endotoxemia in humans. Am J Physiol Endocrinol Metab 286: E766-E772. doi:10.1152/ajpendo.00468.2003. PubMed: 14722028. 14722028 10.1152/ajpendo.00468.2003

[15] Vila G, Maier C, Riedl M, Nowotny P, Ludvik B et al. (2007) Bacterial endotoxin induces biphasic changes in plasma ghrelin in healthy humans. J Clin Endocrinol Metab 92: 3930-3934. doi:10.1210/jc.2007-1194. PubMed: 17666475. 17666475 10.1210/jc.2007-1194

[16] Berk JL, Hagen JF, Beyer WH, Gerber MJ (1970) Hypoglycemia of shock. Ann Surg 171: 400-408. doi:10.1097/00000658-197003000-00013. PubMed: 4906595. 4906595 10.1097/00000658-197003000-00013 PMC1396921

[17] Naylor JM, Kronfeld DS (1985) In vivo studies of hypoglycemia and lactic acidosis in endotoxic shock. Am J Physiol 248: E309-E316. PubMed: 3883802. 3883802 10.1152/ajpendo.1985.248.3.E309

[18] Heuer JG, Sharma GR, Zhang T, Ding C, Bailey DL et al. (2006) Effects of hyperglycemia and insulin therapy on outcome in a hyperglycemic septic model of critical illness. J Trauma 60: 865-872. doi:10.1097/01.ta.0000215565.29846.ab. PubMed: 16612310. 16612310 10.1097/01.ta.0000215565.29846.ab

[19] Hagiwara S, Iwasaka H, Hasegawa A, Asai N, Noguchi T (2009) Hyperglycemia contributes to cardiac dysfunction in a lipopolysaccharide-induced systemic inflammation model. Crit Care Med 37: 2223-2227. doi:10.1097/CCM.0b013e3181a007c6. PubMed: 19487929. 19487929 10.1097/CCM.0b013e3181a007c6

[20] Alonso LC, Watanabe Y, Stefanovski D, Lee EJ, Singamsetty S et al. (2012) Simultaneous measurement of insulin sensitivity, insulin secretion and the disposition index in conscious unhandled mice. Obesity (Silver Spring) 20: 1403-1412. doi:10.1038/oby.2012.36. 22331130 10.1038/oby.2012.36 PMC3378770

[21] Alonso LC, Yokoe T, Zhang P, Scott DK, Kim SK et al. (2007) Glucose infusion in mice: a new model to induce beta-cell replication. Diabetes 56: 1792-1801. doi:10.2337/db06-1513. PubMed: 17400928. 17400928 10.2337/db06-1513 PMC2921922

[22] Ayala JE, Bracy DP, McGuinness OP, Wasserman DH (2006) Considerations in the design of hyperinsulinemic-euglycemic clamps in the conscious mouse. Diabetes 55: 390-397. doi:10.2337/diabetes.55.02.06.db05-0686. PubMed: 16443772. 16443772 10.2337/diabetes.55.02.06.db05-0686

[23] Bergman RN, Phillips LS, Cobelli C (1981) Physiologic evaluation of factors controlling glucose tolerance in man: measurement of insulin sensitivity and beta-cell glucose sensitivity from the response to intravenous glucose. J Clin Invest 68: 1456-1467. doi:10.1172/JCI110398. PubMed: 7033284. 7033284 10.1172/JCI110398 PMC370948

[24] Maedler K, Sergeev P, Ris F, Oberholzer J, Joller-Jemelka HI et al. (2002) Glucose-induced beta cell production of IL-1beta contributes to glucotoxicity in human pancreatic islets. J Clin Invest 110: 851-860. doi:10.1172/JCI15318. PubMed: 12235117. 12235117 10.1172/JCI15318 PMC151125

[25] Mehta VK, Hao W, Brooks-Worrell BM, Palmer JP (1994) Low-dose interleukin 1 and tumor necrosis factor individually stimulate insulin release but in combination cause suppression. Eur J Endocrinol 130: 208-214. doi:10.1530/eje.0.1300208. PubMed: 8130898. 8130898 10.1530/eje.0.1300208

[26] Sauter NS, Schulthess FT, Galasso R, Castellani LW, Maedler K (2008) The antiinflammatory cytokine interleukin-1 receptor antagonist protects from high-fat diet-induced hyperglycemia. Endocrinology 149: 2208-2218. doi:10.1210/en.2007-1059. PubMed: 18239070. 18239070 10.1210/en.2007-1059 PMC2734491

[27] Wei Y, Chen K, Whaley-Connell AT, Stump CS, Ibdah JA, et al (2008) Skeletal muscle insulin resistance: role of inflammatory cytokines and reactive oxygen species. Am J Physiol Regul Integr Comp Physiol 294: R673-680 10.1152/ajpregu.00561.2007 18094066

